# A Neural Network Model of Lexical-Semantic Competition During Spoken Word Recognition

**DOI:** 10.3389/fnhum.2021.700281

**Published:** 2021-09-17

**Authors:** Mihaela Duta, Kim Plunkett

**Affiliations:** ^1^Oxford Research Software Engineering Group, Department of Computer Science, University of Oxford, Oxford, United Kingdom; ^2^Oxford University Babylab, Department of Experimental Psychology, University of Oxford, Oxford, United Kingdom

**Keywords:** language, attention, neuro-computational models, visual world task, machine learning, lexical competition, spoken word recognition

## Abstract

Visual world studies show that upon hearing a word in a target-absent visual context containing related and unrelated items, toddlers and adults briefly direct their gaze toward phonologically related items, before shifting toward semantically and visually related ones. We present a neural network model that processes dynamic unfolding phonological representations of words and maps them to static internal lexical, semantic, and visual representations. The model, trained on representations derived from real corpora, simulates this early phonological over semantic/visual preference. Our results support the hypothesis that incremental unfolding of a spoken word is in itself sufficient to account for the transient preference for phonological competitors over both unrelated and semantically and visually related ones. Phonological representations mapped dynamically in a bottom-up fashion to semantic-visual representations capture the early phonological preference effects reported in visual world tasks. The semantic visual preference typically observed later in such a task does not require top-down feedback from a semantic or visual system.

## 1. Introduction

**Figure 1:**
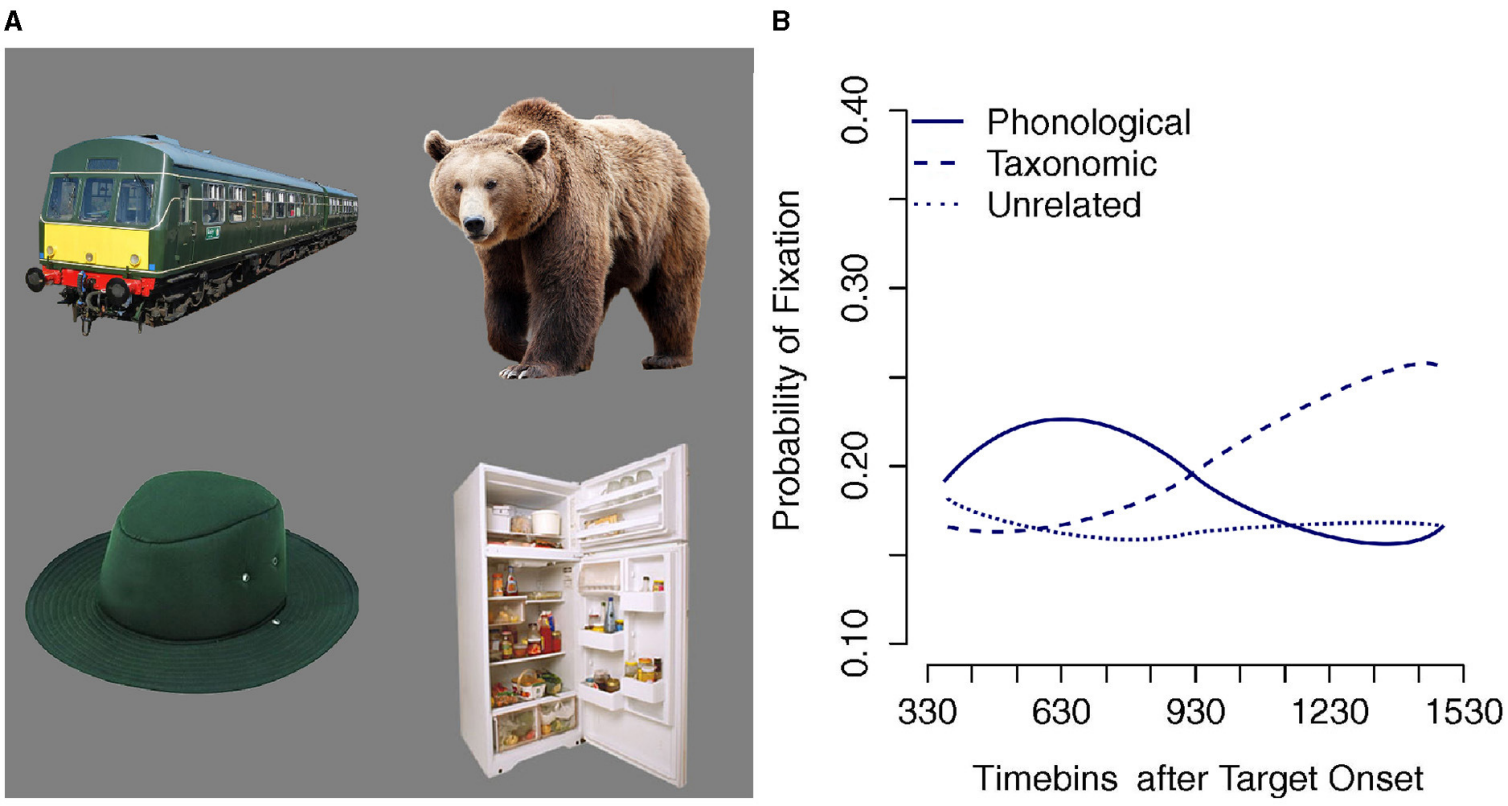
**(A)** Example of the type of display used in visual world tasks (Huettig and McQueen, 2007; Chow et al., 2017). **(B)** Successive fixation of phonological and semantic foils in a 4-picture visual world task by 30-month old toddlers, adapted from Chow et al. (2017).

Upon hearing a spoken word, listeners selectively attend to an item that best matches the word's referent. For example, on seeing a display containing a hat and a bear, listeners hearing the word *trousers* selectively attend to the hat, which is semantically related to the referent of the word *trousers*. Likewise, when hearing *trousers* while presented with a display containing a train and a fridge, they selectively attend to the picture of the train, whose label is phonologically related to *trousers*. In more complex displays such as Figure 1A, which contain both phonological and semantic foils to the referent of *trousers*, listeners exhibit selective attention to both types of foil relative to the unrelated items. Furthermore, listeners selectively and briefly attend to the phonological foil *before* switching attention to the semantically related item. Figure 1B depicts early fixations to phonological foils by 30-month old toddlers within 400 ms of word onset followed by a shift to semantic foils (Chow et al., 2017). Similar results are found with adults, though the initial phonological preference is conditioned by the picture preview time relative to word onset (Huettig and McQueen, 2007).

This pattern of findings is explained by assuming that the listener generates a phonological representation from the unfolding auditory signal and uses this representation to identify the best matching semantic and visual representation generated from the visual input provided by the images. The locus of the match could, in principle, occur at any of the representational levels linking the auditory and visual stimuli: phonological, semantic, or visual. However, the early preference for the phonological foil suggests that the locus of the match resides at the phonological level^1^.

A recent computational model uses a hub-and-spoke, neural network architecture to capture the integration of phonological, semantic and visual information in referent selection in visual world tasks (Smith et al., 2017). The recurrent hub of the model receives inputs from visual and phonological layers, and propagates activity to target semantic and eye layers which themselves feedback activity to the hub. Using an artificially constructed corpus, the model successfully replicates rhyme effects, e.g., when listeners hear *coat*, they selectively attend to the picture of a boat in the absence of a picture of a coat (Allopenna et al., 1998).

Smith et al. (2017) argue that the close integration of visual, phonological, and semantic information in the hub is central to the model's capacity to capture the phonological rhyme effect observed in visual world tasks. We would argue that a feature of the model also critical for obtaining a preference for rhyming over unrelated items is the persistence of all the discrete phonological segments at the input during processing. The rhyming segment of the word thereby comes to dominate the phonological input as the simulation of a visual world trial proceeds.

In this paper, we explore the hypothesis that incremental unfolding of the spoken word, one phonological segment at a time, is sufficient in itself to account for early phonological preferences of the type depicted in Figure 1B, i.e., a transitory early preference for phonologically related items over *both* semantically and visually related items, as well as unrelated ones, followed by a preference for semantically and visually related items over *both* unrelated and phonologically related ones. We evaluate this hypothesis by constructing a neural network model that processes *only* unfolding phonological representations of words at the input and learns to map these dynamic phonological sequences to corresponding static lexical, semantic and visual representations of the words' referents at the output. In essence, the model can be considered to implement a form of lexical comprehension. Particularly noteworthy aspects of the model include:

- All representations used are “naturalistic” insofar as they have been derived from real corpora; these kinds of representations have been shown to predict behavior in visual world tasks (Huettig et al., 2006).
- The vocabulary is derived from a realistic toddler vocabulary taken from parental questionnaire studies (Hamilton et al., 2000).
- The phonological input consists of dynamic, as opposed to static slotted representations.

To anticipate the findings, our model successfully accommodates the early phonological over semantic/visual preference observed in visual world studies (Huettig and McQueen, 2007; Chow et al., 2017). However, we do *not* consider this model a complete account of language mediated attention in visual world settings, but rather a tool to explore the power of dynamic phonological representations in guiding our attention to semantically and visually related items.

## 2. Materials and Methods

The software was developed in *Python 3* (Van Rossum and Drake, 2009) using *numpy, scipy, pandas*, and *plotnine* libraries and models were implemented, trained, and simulated with the *pytorch* machine learning framework (Paszke et al., 2019).

### 2.1. Vocabulary

**Figure 2:**
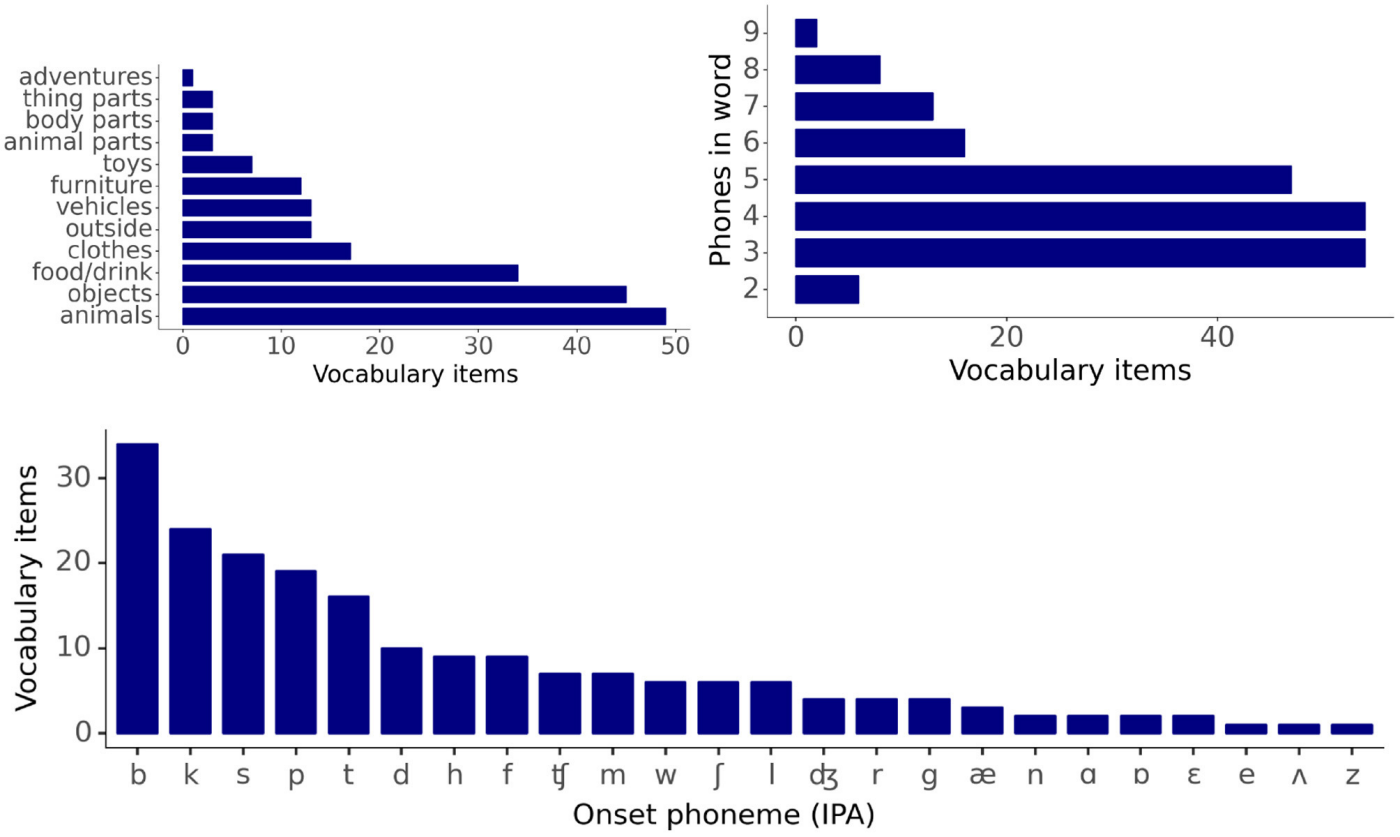
Descriptive statistics for vocabulary items: word category membership, word length distribution, and cohort size distribution.

The vocabulary consists of 200 imageable nouns typically found in the infant lexicon, as documented by the Oxford Communicative Development Inventory data (Hamilton et al., 2000). Vocabulary items come from 12 distinct semantic categories, with a majority (64%) belonging to the categories of animals, food/drink, or household objects. Item labels range in length from 2-phone to 9-phone words, 94.5% of which start with a consonant, and 5.5% with a vowel. The phone inventory of the vocabulary consists of 39 distinct phones, of which 26 are consonants and 13 vowels. Of the 189 items with a consonant onset label, 60% have a cohort of at least 15 items and start with *b, k, p, s*, or *t* phones. Forty-two items in the vocabulary share either three or more onset phones or four or more offset phones (see Supplementary Material). Figure 2 gives distribution plots for category membership, label length, and onset phone identity across the entire vocabulary.

### 2.2. Representations

Each vocabulary item is assigned a dynamic phonological representation of the item label (the unfolding speech pattern) and static semantic, visual, and lexical representations (internal representations).

#### 2.2.1. Phonological and Lexical Representations

Each phone in the inventory is assigned a feature-based distributed binary encoding based on 20 articulatory and phonological features (Karaminis, 2018) (see Supplementary Material for more details). The dynamic phonological representation for each vocabulary item is a matrix composed of the phonological feature representations of its phones in the order in which they appear as the spoken word unfolds, in which each row corresponds to a time step in the unfolding word. To account for phone co-articulation, the transition between two consecutive phone representations is achieved via two intermediate rows between the phonological representations of the two phones, so that the transition between the feature values 1 and 0 consists of two intermediate values of 0.95 and 0.05, and *vice versa*. A segmentation character for which all 20 phonological features are set to 1 was introduced to mark the offset of all labels. The static lexical representation for each vocabulary item is a one-dimensional vector constructed by appending all the feature representations of its phones in the order in which they appear in the word. For both dynamic and lexical representations 10 phone slots are assigned (to accommodate the longest vocabulary item including the segmentation character). Therefore, each dynamic representation is a 20 × 32 feature matrix (10 rows for the phone representations and 12 rows for intermediate co-articulations steps between consecutive phones including ramping up to the first phone and ramping down from the segmentation character) and each lexical representation is a 200 feature vector.

#### 2.2.2. Visual and Semantic Representations

The visual representation for each vocabulary item is derived from the response to an illustration of the item of a *resnet18* deep neural network pre-trained on ImageNet, using the 512-dimensional activation vector for the *avgpool* layer (Deng et al., 2009; He et al., 2016; Paszke et al., 2019). The semantic representations are 100-dimensional word vectors from the GloVe model pre-trained on aggregated global word-word co-occurrence statistics from a 6 billion token corpus composed of the Gigaword5 and Wikipedia 2014 dump (Pennington et al., 2014).

The visual and semantic representation vectors are pre-processed to replace outliers (vector values with a zscore >2) with the median value for the corresponding dimension. Visual representation vectors are further processed using principal component analysis to reduce their dimensionality to 150 (cumulative variance explained: 95%). Both visual and semantic representations are then digitized using two bins (one below and one above the median value for each dimension) to obtain binary vectors.

**Figure 3:**
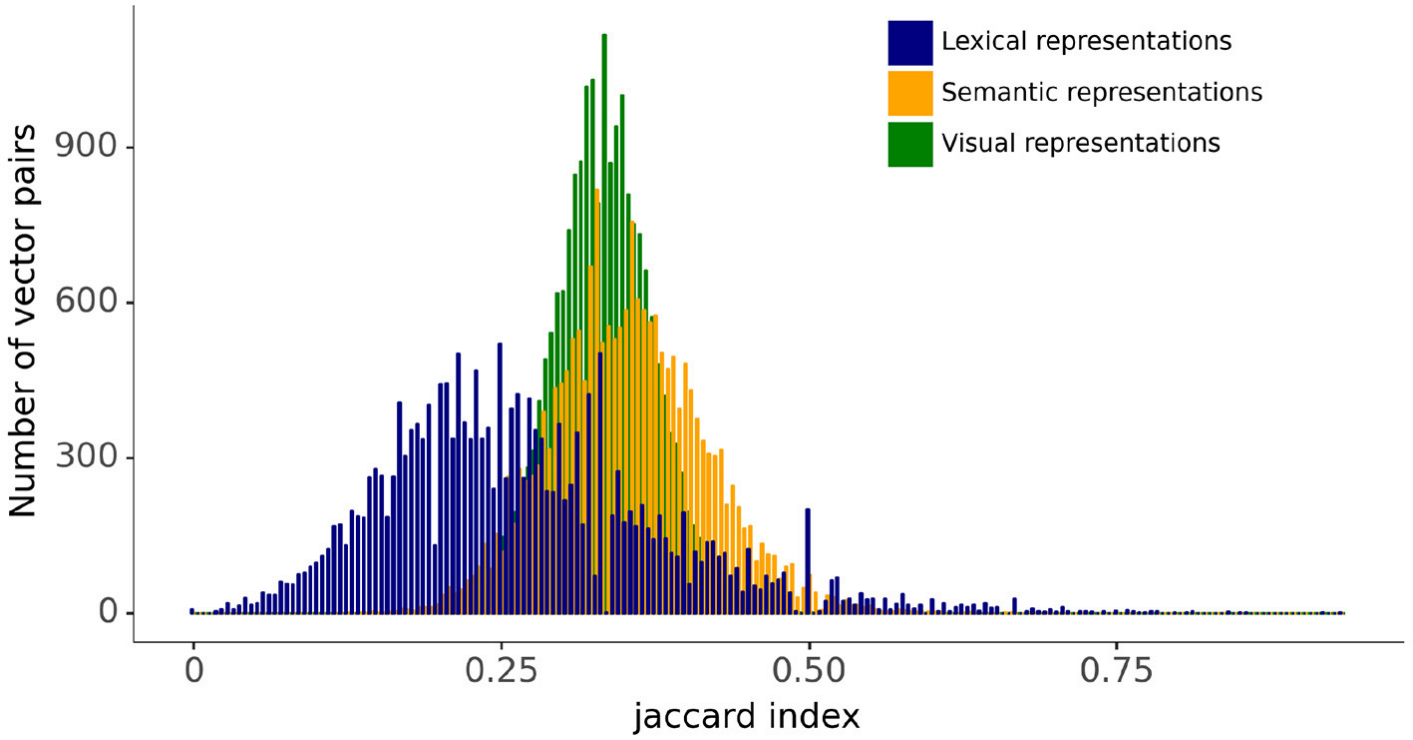
Distributions for Jaccard index between pairs of lexical, semantic, and visual vectors.

The relationships between vectors in the lexical, semantic, and visual representation spaces are evaluated using the Jaccard index, which is a measure of the similarity between two vectors: the larger the Jaccard index, the higher the similarity. The Jaccard index is given by the ratio of the intersection and the union of the components of the two vectors that have a value of 1, therefore giving a measure of the frequency of one-to-one matches between the two vectors. Figure 3 displays the distribution for the Jaccard index for all pairs of lexical, semantic, and visual representation vectors. The figure shows that the distributions of the Jaccard index for pairwise comparisons between semantic and visual vectors are symmetrical and similar in spread. By contrast, the Jaccard index for the pairwise comparisons between lexical forms are more widely distributed with a tailed distribution toward larger index values. This is a reflection of the slotted design of the lexical form representation combined with the fact that words vary in length (see Figure 2). The Jaccard index for the lexical forms captures both the differences in the phonological representations of items' individual phonemes and the differences in word length.

### 2.3. Model Architecture and Training

The model is designed to associate the unfolding of the dynamic phonological representations of the vocabulary items with the corresponding aggregated static semantic, visual, and lexical representations. The model processing cycle for an individual vocabulary item consists of the number of timesteps required to fully unfold the phones in the item's label including the intermediate steps accounting for phone co-articulation and the segmentation character.

**Figure 4:**
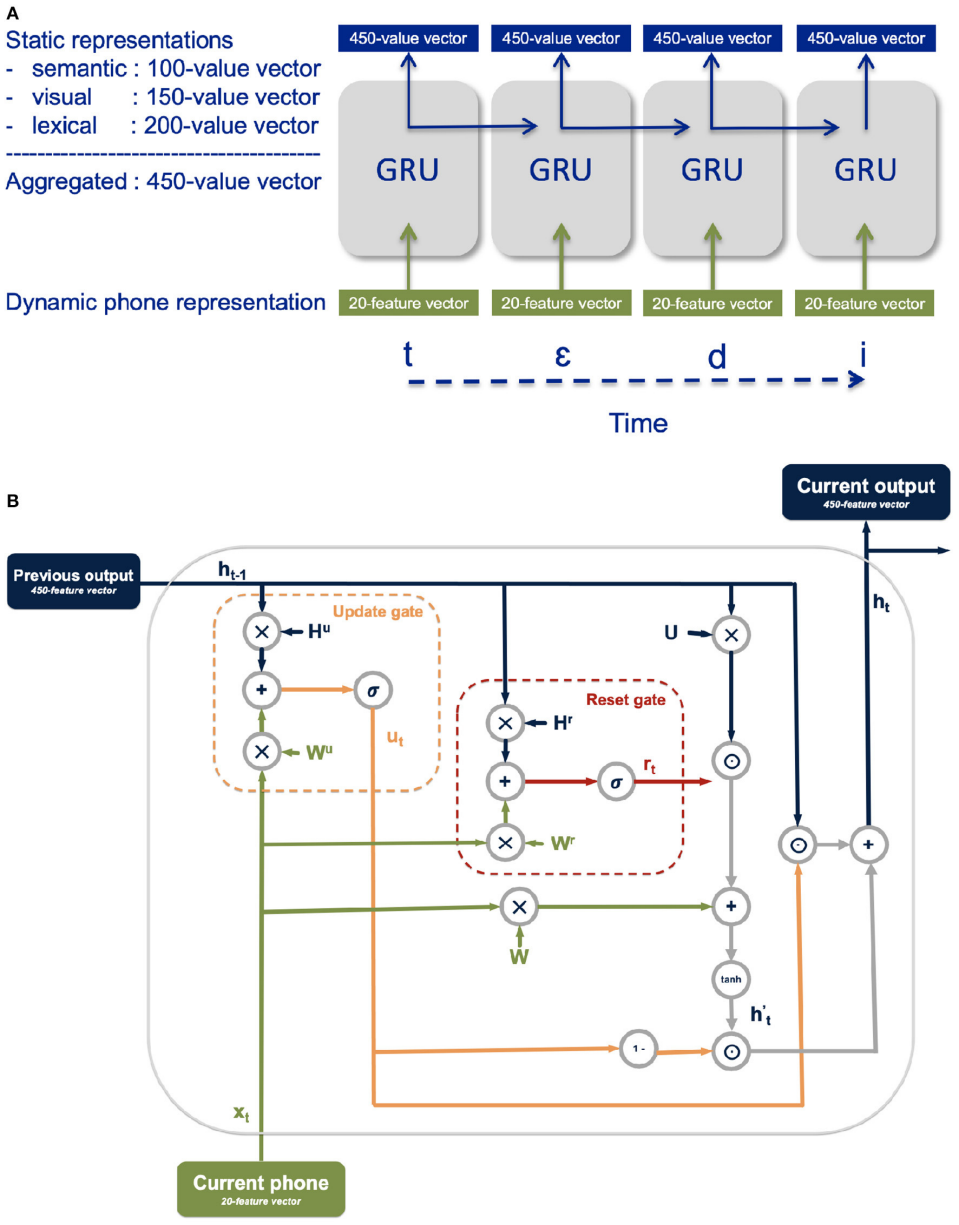
**(A)** Illustration of the model activation at the unfolding of the word *teddy*; intermediate co-articulation timesteps are suppressed in the graphic. **(B)** Detailed illustration of the GRU functionality.

The architecture consists of a gated recurrent unit (GRU) (Cho et al., 2014) whose inputs are a 20-dimensional vector with the encoding of the current phone and whose outputs are a 450-dimensional vector of aggregated semantic, visual, and lexical representations (Figure 4A). A GRU is a recurrent neural network particularly well-suited for processing sequential information, like the unfolding of the phonological representation of a word over time. GRU functionality is achieved via a *reset gate* and an *update gate*, each a trainable vector used in conjunction with the GRU's current input and output from the previous timestep to filter out irrelevant information and retain pertinent information (Figure 4B). The role of the update gate vector is to select the information from the previous processing timestep to be kept for the processing of subsequent timesteps. The role of the *reset gate* is to determine which information from the previous timesteps is irrelevant and therefore does not need to be kept for the processing of subsequent timesteps.

To obtain the update gate vector *u*_*t*_ and the reset gate vector *r*_*t*_ at each time step *t*, the current input *x*_*t*_ and the output from the previous timestep *h*_*t*−1_ are each multiplied with their respective gate weights (*W*^*u*^ and *H*^*u*^ for the update gate and *W*^*r*^, and *H*^*r*^ for the reset gate) and added together before applying a sigmoidal function σ to constrain the vector values between 0 and 1:


(1) $$\begin{array}{l} \begin{array}{c} u_{t}=\sigma \left(W^{u}\times x_{t}+H^{u}\times h_{t-1}\right)\\ r_{t}=\sigma \left(W^{r}\times x_{t}+H^{r}\times h_{t-1}\right) \end{array} \end{array}$$

The reset gate is used in conjunction with the current input and the GRU's output at the previous timestep to select the relevant information from the current time step in the intermediate memory $h_{t}^{\prime }$. First, the current input *x*_*t*_ and the output at the previous timestep *h*_*t*−1_ are both weighted with the *W* and *U* weight vectors, respectively. The element-wise product between the reset vector and the weighted output from the previous timestep is added to the weighted current input before applying a *tanh* function:


(2) $$\begin{array}{l} h_{t}^{\prime }=tanh\left(W\times x_{t}+r_{t}⊙U\times h_{t-1}\right) \end{array}$$

The relevant information from the current timestep is taken as the element-wise product between one minus the update gate vector and the intermediate memory $h_{t}^{\prime }$, while the relevant information from the previous timestep is selected as the element-wise product between the update gate vector and the output at the previous timestep *h*_*t*_. The output of the GRU at the current timestep *h*_*t*_ is then the sum of the relevant information from the current timestep and the relevant information from the previous timestep:


(3) $$h_{t}=u_{t}⊙h_{t-1}+(1-u_{t})⊙h_{t}^{\prime }$$

Training was performed on the entire 200-item vocabulary using batch update and stochastic gradient descent (learning rate: 0.4, momentum: 0.4 and Nesterov momentum enabled, Sutskever et al., 2013). A training trial consisted of the unfolding at the model input of the complete dynamic phonological representation of a word, matched with the corresponding aggregated static semantic, visual, and lexical representations as target. All training trials had the same number of timesteps required to completely unfold the longest label in the vocabulary including the intermediate steps accounting for phone co-articulation and the segmentation character. For shorter labels the model input was padded with zeros from the label offset to the end of the trial. The target semantic, visual, and lexical representations were kept active throughout the duration of the training trial. A training epoch consisted of the presentation to the model of all the 200 training trials corresponding to the entire vocabulary.

### 2.4. Lexical and Semantic-Visual Representations

The model performance for a vocabulary item is given by the model's activation of the item's static representations during the unfolding at the model input of its dynamic phonological representation. The activation of a vocabulary item is given by the Jaccard index between the model output and the item's static representations. The item with the highest Jaccard index is identified as the model's output.

The model output is evaluated separately for the aggregated semantic-visual representations and the lexical representation. The model is considered to have learned a vocabulary item if, at the offset of the unfolding of the item's dynamic phonological representation, the model's highest activation is for that item's target semantic-visual representations. This is equivalent to the model output being closest to that item in the semantic-visual representation space. Similarly, the model is considered to produce the correct lexical output for a vocabulary item if the highest activation is for that item's lexical representation. The progress of learning the semantic-visual and lexical representations of the vocabulary items is evaluated at regular intervals during the training, alongside the impact of word length and cohort size on lexical learning.

### 2.5. Simulating Target-Absent Visual World Contexts

The trained model was evaluated in simulations of visual world trials in which the model activation is calculated for referents in a target-absent context with four potential candidates: a phonologically-related referent, a semantically-related referent, a visually-related referent, and an unrelated referent. At each time step during the unfolding of the target label, the model activation for each candidate referent is calculated as the Jaccard index of the current model output and the referent's aggregated semantic-visual representation. The model is assumed to direct attention to the candidate referent with the highest activation, i.e., the candidate referent whose aggregated semantic-visual representation has the highest Jaccard index compared to the current model output. Note that there is no direct attention mechanism implemented in the model. Instead, the level of activation is used as a proxy for attention, much as eye-fixations are used as a proxy for attention in visual world experiments (Magnuson, 2019).

The selection of the phonologically, semantically, visually, and unrelated candidate trial items for the simulation of the target-absent contexts was done with the following criteria:

- Phonologically related item (PREL): shares the onset phone with the target label, does not share rhyme with the target label and is both semantically and visually unrelated to the target
- Semantically related item (SREL): is semantically related, but visually and phonologically (both onset and rhyme) unrelated to the target
- Visually related item (VREL): is visually related, but semantically and phonologically (both onset and rhyme) unrelated to the target
- Unrelated item (UREL): is phonologically (both onset and rhyme), semantically and visually unrelated to the target

The selection of the semantically and visually related and unrelated items was made using the Jaccard index for the comparison of the target and candidate item representations. An item was considered semantically/visually related or unrelated to the target if the Jaccard index of its semantic/visual representation vector and that of the target was in the top 15th or bottom 15th percentile of all the pairwise comparisons across the vocabulary, respectively. All sets of items that complied with these criteria were used to create a simulation set comprising of 695 trials using 16 target words from seven semantic categories, all with consonant onsets (see Supplementary Material for more details).

## 3. Results

Twenty models^2^ were each trained for 100,000 epochs. This allowed all models to learn both semantic-visual and lexical representations for all vocabulary items. The performance of each model was evaluated every 10,000 epochs during training. First, we consider model performance in learning the target lexical and semantic-visual representations. Second, we evaluate performance of the trained model in target-absent situations, such as those depicted in Figure 1.

### 3.1. Learning Lexical and Semantic-Visual Representations

**Figure 5:**
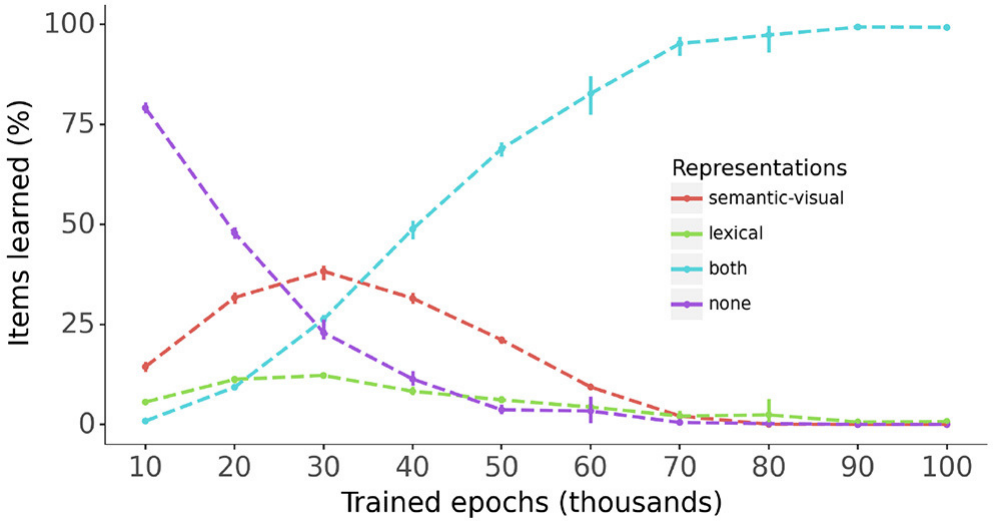
The progress of learning of the semantic-visual and lexical representations across all models. Bars: 95% confidence intervals.

Figure 5 provides a snapshot of model performance every 10,000 epochs during training for the semantic-visual and lexical representations for all vocabulary items across all models. The percentages of items for which both or either constituent semantic-visual and lexical representations are correctly mapped are plotted alongside the percentage of items where neither constituent is correctly mapped. The figure shows a steady increase of the percentage of items for which both the semantic-visual and lexical representations are learned, reaching 100% at end of the training. Interestingly, during the early stages of training (<30,0000 epochs), for a substantial proportion of items (up to 40%) only the phonological → semantic-visual mapping has been mastered, and to a much lesser extent, the phonological → lexical mapping. This amounts to the models learning only the meaning associations or the lexical associations for these items, respectively. The models therefore appear to learn the mapping to some of the semantic-visual or lexical representations, before learning the mapping to both. One might reasonably suppose that learning a word involves learning the representations of both constituents. Figure 5 also shows that the models exhibit accelerated learning of both constituents (as opposed to learning only one of them) between 30,000 and 50,000 epochs.

**Figure 6:**
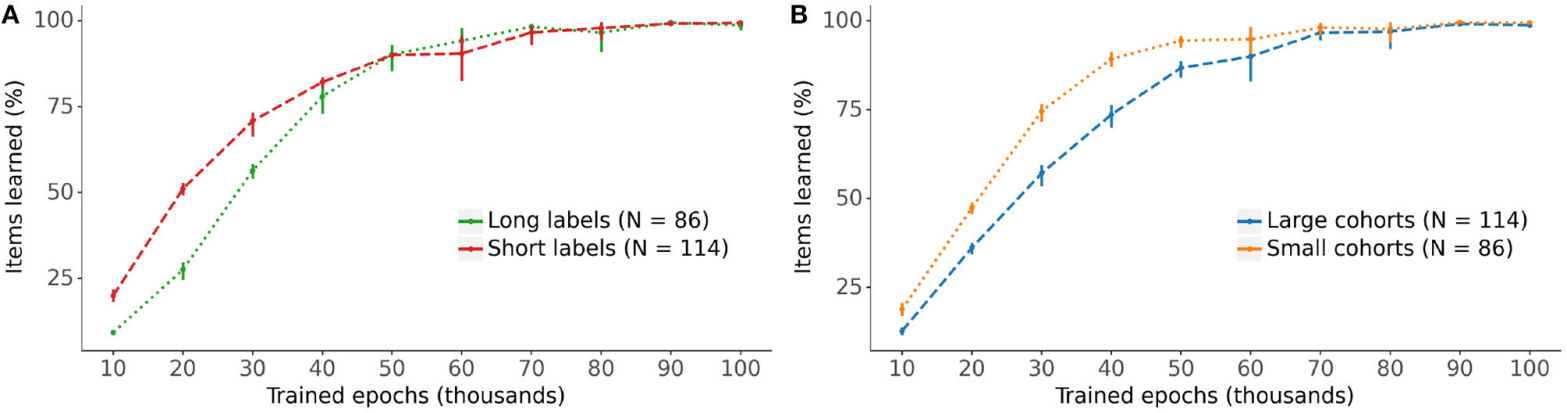
The impact of **(A)** label length and **(B)** cohort size on the learning of the lexical representation. Long labels have 5 phonemes or more, large cohorts contain 15 items or more (see Figure 2). Bars: 95% confidence intervals.

Figure 6 displays the models' progress in learning the lexical representations for the vocabulary items split by label length (long labels are defined as 5 phonemes or more) and cohort size (large cohorts contain 15 items or more). The learning curves indicate a clear advantage in the rate of learning for lexical representations with short labels and small cohorts over those with longer labels and larger cohorts, particularly during the earlier stages of training.

**Figure 7:**
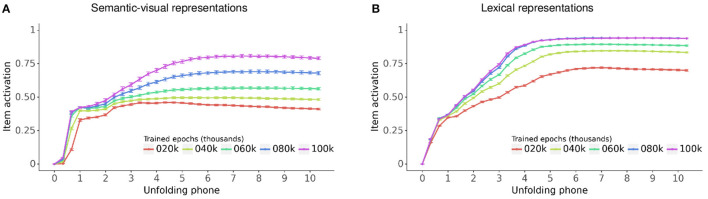
Grand average of the activation time course for vocabulary items as their label unfolds, evaluated every 20,000 epochs during training for **(A)** semantic-visual representations and **(B)** lexical representations. Bars: 95% confidence interval.

Figure 7 displays the activation of the semantic-visual and lexical representations for vocabulary items as their labels unfold at the model input, evaluated every 20,000 epochs during training. The horizontal axis indicates the simulation timesteps for the unfolding of the phones in the item label and the vertical axis is the activation grand average across all models and vocabulary items. Figure 7 shows that as the label unfolds, activations for the item's semantic-visual and lexical representations both steadily increase toward an asymptotic level of item recognition. This level steadily increases across training, as the models gradually fine tune their mastering of the vocabulary items. Throughout training and also for fully trained models the asymptotic level of item recognition is higher for lexical representations compared to semantic-visual representations. This reflects the differences between the distributions of the pairwise Jaccard index within the lexical and semantic-visual representational spaces, which in turn reflects the distribution of the lexical, semantic and visual similarities of the items. The lexical representations are more widely distributed because lexical forms within the vocabulary are quite different from each other aside from occasional random overlap. By contrast, the semantic and visual representations are less widely distributed, which reflects the non-random semantic and visual similarities between the items. This means that when the models recognize a lexical form they do so with higher accuracy than the recognition of the semantic-visual target.

**Figure 8:**
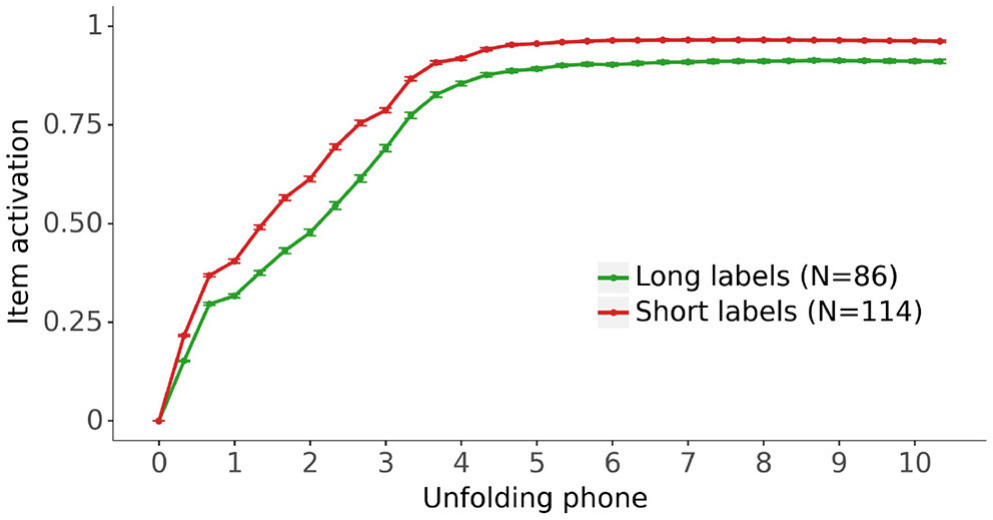
The impact of label length on the grand average activation for the fully trained models of the lexical representation for the vocabulary items as their label unfolds. Long labels have 5 phonemes or more (see Figure 2). Bars: 95% confidence intervals.

Figure 8 displays the activation of the lexical representation for the fully trained models for vocabulary items split by the length of their label. As in Figure 6, long labels are defined as five phonemes or more. Figure 8 shows that the activations of the items with short labels are higher than the activations of the items with long labels, indicating that the models learn better the lexical representations of items with short labels.

### 3.2. Simulating Target-Absent Visual World Contexts

**Figure 9:**
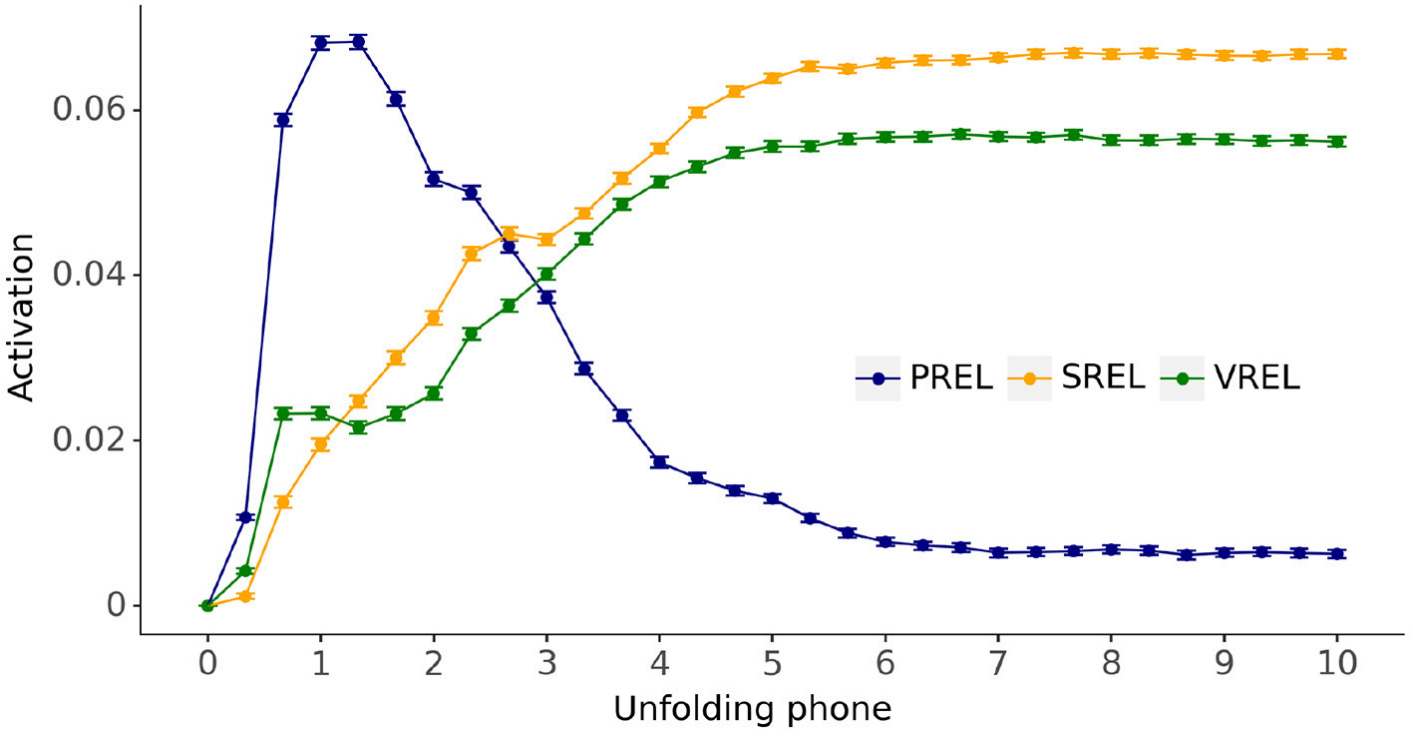
Grand average semantic-visual activation time courses relative to the activation of unrelated distractors (UREL) for phonological candidates (PREL), semantic candidates (SREL), and visual candidates (VREL), evaluated for the fully trained models across all models and simulation trials. Error bars: 95% confidence intervals.

Next, we evaluate the model's capacity to activate semantic-visual representations that are phonologically-related, semantically-related or visually-related to the input, as described in section 2.5. In other words, can the model mimic the target-absent behavior produced by adults and young toddlers in visual world tasks (see Figure 1)? In particular, does the model show an initial preference for phonologically-related items over semantically- or visually-related items as the word unfolds at the input? Figure 9 plots the outcome of simulating the fully trained models in the target-absent visual world contexts. The horizontal axis is simulation timesteps for the unfolding of the phones in the target label and the vertical axis is the grand average of semantic-visual activations for the phonologically-related, semantically-related, and visually-related candidates relative to the activation of the unrelated candidate. The relative activation is calculated by subtracting the activation of the unrelated candidate from the activations of interest for every simulated trial.

Figure 9 shows that activations for the phonologically related candidates are substantially larger than any other activation earlier on in the unfolding of the input label, shifting to larger activations for semantically and visually related candidates later in the trial. Semantically-related candidates maintain a minor advantage over visually-related candidates for the overwhelming majority of simulation timesteps.

## 4. Discussion

The research reported in this paper evaluates the proposal that incremental unfolding of a spoken word is in itself sufficient to account for the pattern of referent selection observed in target-absent trials in visual world tasks containing phonologically related, semantically related, and visual related candidates. We evaluate this proposal with a neural network model designed to map dynamic phonological inputs to static semantic-visual and lexical representations via gated recurrent units (see Figure 4).

### 4.1. Model Learning

Twenty trained models each successfully learned the entire set of 200 vocabulary items. Successful performance is clearly illustrated in Figure 5 which shows that all target lexical and semantic-visual representations are learnt within 100,000 epochs of training. *En route* to mastery of the training set, the model learns some of the semantic-visual representations of the vocabulary items in the absence of learning their lexical representations, and to a lesser extent *vice versa*. This pattern of behavior in the model is akin to that observed in human infants: they may learn the meaning of a word without necessarily having a lexical representation for the same word (Tincoff and Jusczyk, 2012), and they may learn a lexical representation for a word without knowing its meaning (Saffran et al., 1996). However, from 40,000 epochs of training onward, the models master both lexical and semantic-visual target representations for each vocabulary item. This occurs during a period of accelerated vocabulary learning, a feature that resembles vocabulary growth in toddlers during the second year of life. Neuro-computational models of this kind robustly show patterns of nonlinear learning across a range of learning algorithms. However, it remains unclear to what extent such mechanisms are akin to the mechanisms underlying infant behavior. Simulating the model on target items at regular intervals during training also exhibited the expected behavior that lexical and semantic-visual representations become increasingly robust, as indexed by the higher levels of target activation for vocabulary items during the course of training, illustrated in Figure 7.

Model behavior during learning also aligned with aspects of performance of human behavior. In particular, the model exhibited effects of word length and cohort size during the course of learning, whereby shorter labels tend to be learnt before longer labels, and vocabulary items with smaller cohorts are learnt before those with larger cohorts, as illustrated in Figure 6. Although we are unaware of any studies specifically investigating the relation between vocabulary growth and word cohort size, some studies of early lexical development report a deleterious effect of similar sounding words on vocabulary development and lexical processing. In a word learning study with 19-month olds, Swingley and Aslin (2007) reported that phonological similarity with an already known word interferes with novel word learning, suggesting that word cohorts interact with vocabulary development. In a study using picture priming with 24-month olds, Mani and Plunkett (2011) found increased target looking for targets with small cohorts compared to target with large cohorts, a finding which the authors interpret as evidence for an interference effect of the cohort on the processing of target label and item recognition.

### 4.2. Target-Absent Trials

The trained models were simulated in target-absent visual world contexts in which the model activations for the four candidate referents—either unrelated to the referent of the unfolding word, or phonologically, semantically, or visually related to it—are continuously estimated. The activation is the estimated Jaccard index of the comparison between the current model output and the semantic-visual representations of all the candidate referents. Figure 9 depicts a clear early higher activation of the phonological candidate followed by a shift in favor of the semantic and visual candidates later in the trial. We interpret these activations as an early preference for the phonological candidate in a target-absent visual world trial, followed by a later preference for the semantic and visual candidates. These results confirm our proposal that a dynamic unfolding phonological input is sufficient to generate an initial preference for the phonological competitor over *both* semantic and visual competitors in a visual world task.

We now turn to the issue of why our model exhibits an early phonological preference over a semantic-visual preference. Upon ‘hearing’ the onset phone of a word, the model output migrates to the region of the semantic-visual space consistent with the current phonological input. In a target-absent visual world context this is bound to be toward the representation of the phonological competitor—if one is present—which is the only candidate consistent with the onset phone. Therefore, the phonological candidate has the highest activation. However, as the input word unfolds over time, the region of semantic-visual space consistent with the phonological input shifts. The model has been trained to associate *words unfolding toward complete forms* with corresponding semantic-visual and lexical representations: the more of the word the model “hears,” the more its semantic-visual outputs shift toward the semantic-visual associates of the input word. Hence, the models favors phonological competitors before semantic-visual competitors in a target-absent visual world context. The model therefore predicts that in such a task where the scene also contains a phonological onset competitor, unambiguous identification of the target would be delayed relative to a scene that did not contain such a competitor. Evidence for such a delay has been reported in infant word recognition experiments. When 24-month-olds were presented with a display containing a phonological onset competitor (doll-dog), their target responses were delayed but not when the pictures' labels rhymed (doll-ball) (Swingley et al., 1999).

It is worth noting that our model architecture does not permit feedback of activity from the semantic-visual representations to the phonological representations. In other words, there is no “implicit naming” of the stimuli in the visual world trial simulations reported: the model does not generate phonological representations from semantic-visual representations. A corollary of this feature is that the locus of the match between auditory and visual stimuli in a visual world task lies at the semantic-visual level, not at the phonological level. This built-in assumption of the model is at odds with the claim that reducing picture preview time in a visual world task can eliminate early phonological preferences (see Huettig and McQueen, 2007). However, we note a growing body of empirical evidence that an extended picture preview time is not required to observe an early phonological preference effect in visual world tasks (Rigler et al., 2015; Villameriel et al., 2019). These recent findings point to the possibility that other task demands that highlight semantic competitors may suppress phonological effects during referent identification.

Some forms of semantic feedback, such as that implemented in Smith et al. (2017), may serve to eliminate early phonological preferences in visual world tasks in certain circumstances, such as those reported by Huettig and McQueen (2007). In this case, identification of the neuro-computational mechanism(s) responsible for controlling the presence/absence of the widely-reported phonological effects would be required. We speculate that growth in *top-down* connectivity from semantic representations, perhaps through the emergence and consolidation of the lexical-semantic system, may permit semantic-visual representations to modulate the *bottom-up* phonological processes as implemented in the current model.

We conclude that phonological representations mapped dynamically in a *bottom-up* fashion to semantic-visual and lexical representations are *sufficient* to capture the early phonological preference effects reported in a target-absent visual world task. The semantic-visual preference observed later in such a trial does not require *top-down* feedback from a semantic or visual system. We do not claim that such top-down connections do not exist. Indeed, we would expect a proper computational account of the visual world task to include such resources. Our strategy has been to seek to minimize the computational resources needed to account for the phenomenon at hand. We suppose that incremental development of these resources is the best way to achieve understanding of visual world processes.

## Data Availability Statement

The raw data supporting the conclusions of this article will be made available by the authors, without undue reservation.

## Author Contributions

MD designed and implemented the models, derived and processed the input and output representations, ran the simulations, processed and analyzed the data, and wrote the article. KP was responsible for project conception and contributed to the data analysis and writing of the article. Both authors approved the submitted version.

## Funding

This work was funded by the Leverhulme Trust grant RPG-2017-307 to KP.

## Conflict of Interest

The authors declare that the research was conducted in the absence of any commercial or financial relationships that could be construed as a potential conflict of interest.

## Publisher's Note

All claims expressed in this article are solely those of the authors and do not necessarily represent those of their affiliated organizations, or those of the publisher, the editors and the reviewers. Any product that may be evaluated in this article, or claim that may be made by its manufacturer, is not guaranteed or endorsed by the publisher.

## Footnotes

^1^Huettig and McQueen (2007) also point out that removal of the picture preview phase in this task obliterates the early phonological preference, presumably because participants don't have time to generate the phonological codes for the images.

^2^The models all have the same architecture but for each model the training is started from a different random initial state.
