# Supplementary material for: A Neural Network Model of Lexical-Semantic Competition During Spoken Word Recognition
Source: Front Hum Neurosci. 2021 Sep 17;15:700281. doi: 10.3389/fnhum.2021.700281 (PMC8484523; doi:10.3389/fnhum.2021.700281)
Supplement: Supplementary file 1 [file Data_Sheet_1.pdf]

frontiers

A Neural Network Model of Lexical-Semantic Competition during Spoken Word Recognition Supplementary material

Mihaela Duta and Kim Plunkett

Table S1: Phone encodings

Phone	Consonant	Obstruent	Sonorant	Voiced	Plosive	Continuant	Nasal	Lateral	Rhotic	Strident	Labial	Coronal	Dorsal	Glottal	Distributed	High	Mid	Low	Retracted	Long
p	1	1	0	0	1	0	0	0	0	0	1	0	0	0	0	0	0	0	0	0
b	1	1	0	1	1	0	0	0	0	0	1	0	0	0	0	0	0	0	0	0
t	1	1	0	0	1	0	0	0	0	0	0	1	0	0	0	0	0	0	0	0
d	1	1	0	1	1	0	0	0	0	0	0	1	0	0	0	0	0	0	0	0
k	1	1	0	0	1	0	0	0	0	0	0	0	1	0	0	0	0	0	0	0
g	1	1	0	1	1	0	0	0	0	0	0	0	1	0	0	0	0	0	0	0
m	1	0	1	1	0	0	1	0	0	0	1	0	0	0	0	0	0	0	0	0
n	1	0	1	1	0	0	1	0	0	0	0	1	0	0	0	0	0	0	0	0
ŋ	1	0	1	1	0	0	1	0	0	0	0	0	1	0	0	0	0	0	0	0
f	1	1	0	0	0	1	0	0	0	0	1	0	0	0	0	0	0	0	0	0
v	1	1	0	1	0	1	0	0	0	0	1	0	0	0	0	0	0	0	0	0
θ	1	1	0	0	0	1	0	0	0	0	0	1	0	0	0	0	0	1	0	0
ð	1	1	0	1	0	1	0	0	0	0	0	1	0	0	0	0	0	1	0	0
s	1	1	0	0	0	1	0	0	0	1	0	1	0	0	0	0	0	1	0	0
z	1	1	0	1	0	1	0	0	0	1	0	1	0	0	0	0	0	1	0	0
ʒ	1	1	0	1	0	1	0	0	0	1	0	1	0	0	0	0	0	0	0	0
ʃ	1	1	0	0	0	1	0	0	0	1	0	1	0	0	0	0	0	0	0	0
r	1	0	1	1	0	0	0	0	1	0	0	1	0	0	0	0	0	0	0	0
h	1	1	0	0	0	1	0	0	0	0	0	0	0	1	0	0	0	0	0	0
j	1	0	1	1	0	0	0	0	0	0	0	1	0	0	0	1	0	0	0	0
l	1	0	1	1	0	0	0	1	0	0	0	1	0	0	0	0	0	0	0	0
w	1	0	1	1	0	0	0	0	0	0	1	0	1	0	0	1	0	0	0	0
ʧ	1	1	0	0	1	0	0	0	0	1	0	1	0	0	1	1	0	0	0	0
ʤ	1	1	0	1	1	0	0	0	0	1	0	1	0	0	1	1	0	0	0	0
æ	0	0	1	1	0	0	0	0	0	0	0	1	0	0	0	0	0	1	0	0
ɒ	0	0	1	1	0	0	0	0	0	0	0	0	1	0	0	0	0	1	0	0
ɒː	0	0	1	1	0	0	0	0	0	0	0	0	1	0	0	0	0	1	0	1
ɒ	0	0	1	1	0	0	0	0	0	0	1	0	1	0	0	0	0	1	0	0
ɛ	0	0	1	1	0	0	0	0	0	0	0	1	0	0	0	0	1	0	1	0
ɛː	0	0	1	1	0	0	0	0	0	0	0	1	0	0	0	0	1	0	1	1
e	0	0	1	1	0	0	0	0	0	0	0	1	0	0	0	0	1	0	0	0

ɜ	0	0	1	1	0	0	0	0	0	0	0	0	1	0	0	0	1	0	0	1
i	0	0	1	1	0	0	0	0	0	0	0	1	0	0	0	1	0	0	0	0
iː	0	0	1	1	0	0	0	0	0	0	0	1	0	0	0	1	0	0	0	1
ɪ	0	0	1	1	0	0	0	0	0	0	0	1	0	0	0	1	0	0	1	0
ɔ	0	0	1	1	0	0	0	0	0	0	1	0	1	0	0	0	1	0	1	0
ɔ	0	0	1	1	0	0	0	0	0	0	1	0	1	0	0	0	1	0	1	1
ə	0	0	1	1	0	0	0	0	0	0	0	0	1	0	0	0	1	0	1	0
uː	0	0	1	1	0	0	0	0	0	0	1	0	1	0	0	1	0	0	0	1
ʊ	0	0	1	1	0	0	0	0	0	0	1	0	1	0	0	1	0	0	1	0
ʌ	0	0	1	1	0	0	0	0	0	0	0	0	1	0	0	0	1	0	1	0

Table S2: Lexicon items

Label	IPA	CDI category
acorn	eɪkɔn	outside
ambulance	æmbjʊləns	vehicles
ant	ænt	animals
apple	æpəl	food/drink
backpack	bækpæk	objects
bag	bæg	objects
ball	bɔl	toys
balloon	bəluːn	toys
banana	bənɒːnə	food/drink
bath	bɒːθ	furniture
beach	biːʧ	outside
bear	bɛə	animals
bed	bɛd	furniture
bee	biː	animals
belt	bɛlt	clothes
bib	bɪb	clothes
bike	bɒɪk	vehicles
bin	bɪn	objects
bird	bɜd	animals
biscuit	bɪskɪt	food/drink
blackberry	blækbəri	food/drink
blanket	blænkət	objects
block	blɒk	toys
blueberry	bluːbəri	food/drink
boat	bəʊt	vehicles
boot	buːt	clothes
bottle	bɒtəl	objects
bowl	bəʊl	objects
box	bɒks	objects
bread	brɛd	food/drink
broom	bruːm	objects

brush	brʌʃ	objects
bubble	bʌbl	toys
bucket	bʌkɪt	toys
bunny	bʌni	animals
bus	bʌs	vehicles
butter	bʌtə	food/drink
butterfly	bʌtəflɒɪ	animals
camel	kæməl	animals
camera	kæmərə	objects
car	kɒː	vehicles
carrot	kærət	food/drink
cat	kæt	animals
caterpillar	kætəpɪlə	animals
cereal	sɪərɪəl	food/drink
chair	ʧɛə	furniture
cheese	ʧːz	food/drink
cherry	ʧɛri	food/drink
chicken	ʧɪkɪn	animals
chips	ʧɪps	food/drink
chocolate	ʧɒkəlɪt	food/drink
church	ʧɛːʧ	outside
clock	klɒk	objects
coat	kəʊt	clothes
coffee	kɒfi	food/drink
comb	kəʊm	objects
computer	kəmpjuːtə	objects
cooker	kʊkə	furniture
cookie	kʊki	food/drink
cot	kɒt	furniture
cow	kɒʊ	animals
crab	kræb	animals
crane	kreɪn	vehicles
cucumber	kjuːkʌmbə	food/drink
cup	kʌp	objects
cushion	kʊʃən	objects
deer	dɪə	animals
digger	dɪgə	vehicles
dinosaur	dɒɪnəʊsɔ	adventures
dog	dɒg	animals
dolphin	dɒlfɪn	animals
donkey	dɒŋki	animals
door	dɔ	furniture
drawer	drɔ	furniture
dress	drɛs	clothes
duck	dʌk	animals

egg	ɛg	food/drink
elephant	ɛlɪfənt	animals
face	feɪs	body parts
feather	fɛðə	animal parts
fish	fɪʃ	animals
flag	flæg	outside
flower	flɒʊə	outside
fork	fɔk	objects
fridge	frɪʤ	furniture
frog	frɒg	animals
giraffe	ʤɪrɒːf	animals
glass	glɒːs	objects
glasses	glɒːsəz	clothes
goose	guːs	animals
grape	greɪp	food/drink
hair	hɛə	body parts
hammer	hæmə	objects
handbag	hændbæg	objects
handle	hændl	thing parts
hat	hæt	clothes
headphones	hɛdfəʊnz	objects
helicopter	hɛlɪkɒptə	vehicles
hoover	huːvə	objects
horse	hɔs	animals
iron	ɒɪən	objects
jug	ʤʌg	objects
juice	ʤuːs	food/drink
jumper	ʤʌmpə	clothes
key	kiː	objects
keyboard	kiːbɔd	objects
kitten	kɪtən	animals
koala	kəʊɒːlə	animals
lamb	læm	animals
lamp	læmp	objects
laptop	læptɒp	objects
lemon	lɛmən	food/drink
lion	lɒɪən	animals
lizard	lɪzəd	animals
meat	miːt	food/drink
melon	mɛlən	food/drink
milk	mɪlk	food/drink
money	mʌni	objects
monkey	mʌŋki	animals
mouse	mɒʊs	animals
mug	mʌg	objects

nappy	næpi	clothes
necklace	nɛklɪs	clothes
octopus	ɒktəpəs	animals
orange	ɒrɪnʤ	food/drink
owl	ɒʊl	animals
panda	pændə	animals
park	pɒːk	outside
pasta	pæstə	food/drink
pear	pɛə	food/drink
peas	piːz	food/drink
pen	pɛn	toys
penguin	pɛŋgwɪn	animals
phone	fəʊn	objects
picture	pɪkʧə	objects
pig	pɪg	animals
pillow	pɪləʊ	objects
pizza	piːtsə	food/drink
plane	pleɪn	vehicles
plant	plɒːnt	objects
plate	pleɪt	objects
pony	pəʊni	animals
porridge	pɒrɪʤ	food/drink
printer	prɪntə	objects
puppy	pʌpi	animals
pyjamas	pəʤɒːməz	clothes
rabbit	ræbɪt	animals
raisins	reɪzɪŋ	food/drink
remote	rɪməʊt	objects
rocket	rɒkɪt	vehicles
scissors	sɪzəz	objects
seal	siːl	animals
sheep	ʃiːp	animals
shell	ʃɛl	animal parts
shirt	ʃɛːt	clothes
shoe	ʃuː	clothes
shop	ʃɒp	outside
shorts	ʃɔts	clothes
sink	sɪŋk	furniture
snail	sneɪl	animals
soap	səʊp	objects
sock	sɒk	clothes
sofa	səʊfə	furniture
spade	speɪd	outside
speakers	spiːkəz	objects
spider	spɒɪdə	animals

spoon	spuːn	objects
squirrel	skwɪrəl	animals
star	stɒː	outside
stick	stɪk	outside
strawberry	strɔbəri	food/drink
sun	sʌn	outside
swan	swɒn	animals
sweets	swiːts	food/drink
swing	swɪŋ	outside
switch	swɪʧ	thing parts
table	teɪbəl	furniture
teddy	tɛdi	toys
tie	tɒɪ	clothes
tiger	tɒɪgə	animals
tissue	tɪʃuː	objects
toast	təʊst	food/drink
tomato	təmɒːtəʊ	food/drink
tooth	tuːθ	body parts
towel	tɒʊəl	objects
tractor	træktə	vehicles
train	treɪn	vehicles
trousers	trɒʊzəz	clothes
truck	trʌk	vehicles
turkey	tɜki	animals
turtle	tɜtəl	animals
tv	tiːviː	furniture
umbrella	ʌmbrɛlə	objects
wall	wɔl	outside
watch	wɒʧ	objects
wheel	wiːl	thing parts
wings	wɪŋz	animal parts
wipes	wɒɪps	objects
worm	wɜm	animals
zebra	ziːbrə	animals

Table S3. Items sharing either three or more onset phones or four or more offset phones.

Label	IPA	Label	IPA	Label	IPA	Label	IPA
bag	bæg	cat	kæt	key	kiː	swing	swɪŋ
beach	biːʧ	caterpillar	kætəpɪlə	keyboard	kiːbɔd	switch	swɪʧ
bee	biː	coat	kəʊt	koala	kəʊɒːlə	tie	tɒɪ
blackberry	blækbəri	comb	kəʊm	lamb	læm	tiger	tɒɪgə
blanket	blænkət	cooker	kʊkə	lamp	læmp	tissue	tɪʃuː
blueberry	bluːbəri	cookie	kʊki	lion	lɒɪən	train	treɪn
boat	bəʊt	crane	kreɪn	plane	pleɪn		
bowl	bəʊl	glass	glɒːs	plate	pleɪt		
butter	bʌtə	glasses	glɒːsəz	shoe	ʃuː		
butterfly	bʌtəflɒɪ	handbag	hændbæg	soap	səʊp		
camel	kæməl	handle	hændl	sofa	səʊfə		
camera	kæmərə	iron	ɒɪən	strawberry	strɔbəri		

Table S4. Target items for PVSU^{MD} simulation trials

Target	IPA	CDI category
bib	bɪb	clothes
carrot	kærət	food/drink
dress	drɛs	clothes
broom	bruːm	objects
swan	swɒn	animals
towel	tɒʊəl	objects
plane	pleɪn	vehicles
keyboard	kiːbɔd	objects
blanket	blænkət	objects
star	stɒː	outside
deer	dɪə	animals
glasses	glɒːsəz	clothes
lemon	lɛmən	food/drink
bubble	bʌbl	toys
bird	bɜd	animals
spoon	spuːn	objects
